# Supplementary material for: Dynamical gene regulatory networks are tuned by transcriptional autoregulation with microRNA feedback
Source: Sci Rep. 2020 Jul 31;10:12960. doi: 10.1038/s41598-020-69791-5 (PMC7395740; doi:10.1038/s41598-020-69791-5)
Supplement: Supplementary file 1 — Supplementary Legends. [file 41598_2020_69791_MOESM1_ESM.docx]

**Supplementary Figures and Table Legends**

**Supplementary Figure 1: Tissue networks are not similar.**

(A) Heatmap of Jaccard indexes between networks based on the TF to Target interactions. MicroRNA interactions were excluded as they are the same for all tissue networks.

(B) Histogram of pairwise comparisons of networks showing the number of comparisons against the Jaccard index (%). Most tissues network pairs have a Jaccard index of ≤ 10%.

**Supplementary Table 1:** Gene ontology enrichment analysis results from GOnet

**Supplementary Table 2:** Cell lines assigned to each tissue

**Supplementary Table 3:** TFs assigned to each tissue
