# Supplementary material for: Dynamical gene regulatory networks are tuned by transcriptional autoregulation with microRNA feedback
Source: Sci Rep. 2020 Jul 31;10:12960. doi: 10.1038/s41598-020-69791-5 (PMC7395740; doi:10.1038/s41598-020-69791-5)
Supplement: Supplementary file 7 — Supplementary Information 5. [file 41598_2020_69791_MOESM7_ESM.docx]

**Dynamical gene regulatory networks are tuned by transcriptional**

**autoregulation with microRNA feedback.**

Thomas G Minchington^1^, Sam Griffiths-Jones^2*^ and Nancy Papalopulu^1*^

**Supplementary Tables**

**Supplementary Table 1**

| **GO term ID** | **GO term definition** | **P** | **P FDR adjusted** | **No. of Genes** | **Genes** |
| --- | --- | --- | --- | --- | --- |
| GO:0048513 | animal organ development | 1.04E-06 | 1.70E-03 | 153 | AR\|ARID1A\|ARID2\|ARNT\|ARRB1\|ATF2\|ATF3\|BATF\|BCL3\|BCL6\|BCOR\|BMI1\|CBFB\|CDK6\|CDX2\|CEBPB\|CEBPD\|CEBPG\|CHD8\|CREB1\|E2F1\|E2F7\|EED\|EGR1\|ELF3\|EOMES\|ESR1\|ETS1\|ETV1\|ETV6\|EZH1\|EZH2\|FLI1\|FOS\|FOSL1\|FOSL2\|FOXA1\|FOXK1\|FOXO1\|FOXP1\|FOXP2\|GATA1\|GATA2\|GATA3\|GATA4\|GRHL2\|HIF1A\|HOXB13\|HSF1\|ID3\|IKZF1\|IRF1\|IRF4\|JMJD6\|KDM1A\|KDM5A\|KDM5B\|KDM6B\|KLF1\|KLF4\|KLF5\|KLF6\|KMT2A\|LHX2\|LYL1\|MAX\|MBD1\|MBD2\|MBD3\|MEF2A\|MEF2C\|MEIS1\|MEIS2\|MITF\|MYC\|MYOD1\|NCOA1\|NCOR2\|NFATC1\|NFIC\|NFKB2\|NKX2-1\|NKX2-2\|NKX3-1\|NOTCH1\|NR2F2\|NRIP1\|NSD2\|ONECUT1\|PAX5\|PAX8\|PBX3\|PDX1\|PGR\|PHF8\|PKNOX1\|PML\|POU2F2\|POU4F2\|PPARG\|PROX1\|RARA\|RBPJ\|RELB\|REST\|RUNX1\|RUNX2\|RUNX3\|RXRA\|SAP30\|SIN3A\|SIX5\|SMAD2\|SMAD3\|SMAD5\|SMARCA4\|SMARCC1\|SNAI2\|SOX11\|SOX2\|SOX6\|SPI1\|SREBF1\|SRF\|STAT1\|STAT3\|SVIL\|T\|TAF1\|TAL1\|TBL1XR1\|TBX21\|TCF12\|TCF3\|TCF7\|TCF7L2\|TEAD4\|TFAP2A\|TFAP2C\|TGIF2\|TLE3\|TP53\|TP63\|TP73\|VDR\|YAP1\|YY1\|ZBTB16\|ZBTB7A\|ZEB1\|ZEB2\|ZHX2\|ZMIZ1 |
| GO:0048731 | system development | 1.19E-06 | 1.70E-03 | 169 | ADNP\|AHR\|AR\|ARID1A\|ARID2\|ARNT\|ARRB1\|ATF1\|ATF2\|ATF3\|BATF\|BCL11A\|BCL3\|BCL6\|BCOR\|BHLHE40\|BMI1\|CBFA2T2\|CBFB\|CDK6\|CDX2\|CEBPB\|CEBPD\|CEBPG\|CHD8\|CREB1\|CUX1\|DPF2\|E2F1\|E2F7\|EED\|EGR1\|ELF3\|EOMES\|ESR1\|ETS1\|ETV1\|ETV6\|EZH1\|EZH2\|FLI1\|FOS\|FOSL1\|FOSL2\|FOXA1\|FOXK1\|FOXO1\|FOXP1\|FOXP2\|GATA1\|GATA2\|GATA3\|GATA4\|GRHL2\|HIF1A\|HOXB13\|HSF1\|ID3\|IKZF1\|IRF1\|IRF4\|JMJD6\|KDM1A\|KDM4C\|KDM5A\|KDM5B\|KDM6B\|KLF1\|KLF4\|KLF5\|KLF6\|KMT2A\|LHX2\|LYL1\|MAX\|MBD1\|MBD2\|MBD3\|MEF2A\|MEF2C\|MEIS1\|MEIS2\|MITF\|MYB\|MYC\|MYOD1\|NBN\|NCOA1\|NCOR2\|NFATC1\|NFE2L2\|NFIC\|NFKB2\|NKX2-1\|NKX2-2\|NKX3-1\|NME2\|NOTCH1\|NR2C2\|NR2F2\|NR2F6\|NRIP1\|NSD2\|ONECUT1\|PAX5\|PAX8\|PBX3\|PDX1\|PGR\|PHF8\|PKNOX1\|PML\|POU2F2\|POU4F2\|PPARG\|PROX1\|RARA\|RBPJ\|RELB\|REST\|RUNX1\|RUNX2\|RUNX3\|RXRA\|SAP30\|SIN3A\|SIX5\|SMAD2\|SMAD3\|SMAD5\|SMARCA4\|SMARCC1\|SNAI2\|SOX11\|SOX2\|SOX6\|SPI1\|SREBF1\|SRF\|STAT1\|STAT3\|SVIL\|T\|TAF1\|TAL1\|TBL1XR1\|TBX21\|TCF12\|TCF3\|TCF7\|TCF7L2\|TEAD4\|TFAP2A\|TFAP2C\|TGIF2\|TLE3\|TP53\|TP63\|TP73\|VDR\|YAP1\|YY1\|ZBTB16\|ZBTB7A\|ZEB1\|ZEB2\|ZHX2\|ZMIZ1\|ZNF24 |
| GO:0007275 | multicellular organism development | 1.61E-05 | 1.54E-02 | 185 | ADNP\|AHR\|AR\|ARID1A\|ARID2\|ARNT\|ARRB1\|ATF1\|ATF2\|ATF3\|BATF\|BCL11A\|BCL3\|BCL6\|BCOR\|BHLHE40\|BMI1\|BRCA1\|CBFA2T2\|CBFB\|CDK6\|CDX2\|CEBPB\|CEBPD\|CEBPG\|CHD8\|CREB1\|CREB3L1\|CREBBP\|CREM\|CUX1\|DPF2\|E2F1\|E2F7\|EBF1\|EED\|EGR1\|ELF3\|EOMES\|ERG\|ESR1\|ETS1\|ETV1\|ETV6\|EZH1\|EZH2\|FLI1\|FOS\|FOSL1\|FOSL2\|FOXA1\|FOXK1\|FOXO1\|FOXP1\|FOXP2\|GATA1\|GATA2\|GATA3\|GATA4\|GFI1B\|GRHL2\|HCFC1\|HIF1A\|HOXB13\|HSF1\|ID3\|IKZF1\|IRF1\|IRF4\|JMJD6\|KDM1A\|KDM4C\|KDM5A\|KDM5B\|KDM6B\|KLF1\|KLF4\|KLF5\|KLF6\|KMT2A\|LHX2\|LMO2\|LYL1\|MAX\|MBD1\|MBD2\|MBD3\|MEF2A\|MEF2C\|MEIS1\|MEIS2\|MITF\|MNT\|MYB\|MYC\|MYOD1\|NBN\|NCOA1\|NCOR2\|NELFA\|NFATC1\|NFE2\|NFE2L2\|NFIC\|NFKB2\|NKX2-1\|NKX2-2\|NKX3-1\|NME2\|NOTCH1\|NR2C2\|NR2F2\|NR2F6\|NRIP1\|NSD2\|ONECUT1\|PAX5\|PAX8\|PBX3\|PDX1\|PGR\|PHF8\|PKNOX1\|PML\|POU2F2\|POU4F2\|POU5F1\|PPARG\|PRDM14\|PROX1\|RAD21\|RARA\|RBPJ\|RELB\|REST\|RUNX1\|RUNX2\|RUNX3\|RXRA\|SAP30\|SIN3A\|SIX5\|SMAD2\|SMAD3\|SMAD5\|SMARCA4\|SMARCC1\|SNAI2\|SOX11\|SOX2\|SOX6\|SPI1\|SREBF1\|SRF\|STAT1\|STAT3\|SVIL\|T\|TAF1\|TAL1\|TBL1XR1\|TBX21\|TCF12\|TCF3\|TCF7\|TCF7L2\|TEAD4\|TFAP2A\|TFAP2C\|TGIF2\|TLE3\|TOP1\|TP53\|TP63\|TP73\|VDR\|YAP1\|YY1\|ZBTB16\|ZBTB7A\|ZEB1\|ZEB2\|ZHX2\|ZMIZ1\|ZNF24 |
| GO:0006357 | regulation of transcription by RNA polymerase II | 3.29E-05 | 2.36E-02 | 247 | ADNP\|AHR\|AR\|ARID1A\|ARID2\|ARID3A\|ARNT\|ARRB1\|ATF1\|ATF2\|ATF3\|ATF7\|BACH1\|BACH2\|BATF\|BCL11A\|BCL3\|BCL6\|BCOR\|BHLHE40\|BMI1\|BRCA1\|BRD3\|BRD4\|CBFA2T2\|CBFB\|CDK6\|CDK8\|CDK9\|CDX2\|CEBPB\|CEBPD\|CEBPG\|CHD1\|CHD8\|CIITA\|CREB1\|CREB3L1\|CREBBP\|CREM\|CTBP2\|CTCFL\|CUX1\|DEK\|DPF2\|E2F1\|E2F7\|EBF1\|EED\|EGLN2\|EGR1\|ELF1\|ELF3\|ELK1\|EOMES\|ERG\|ESR1\|ETS1\|ETV1\|ETV4\|ETV6\|EZH1\|EZH2\|FLI1\|FOS\|FOSL1\|FOSL2\|FOXA1\|FOXK1\|FOXK2\|FOXO1\|FOXP1\|FOXP2\|GATA1\|GATA2\|GATA3\|GATA4\|GFI1B\|GRHL2\|HBP1\|HCFC1\|HDAC8\|HDGF\|HIF1A\|HMBOX1\|HNF4G\|HOXB13\|HSF1\|ID3\|IKZF1\|IRF1\|IRF2\|IRF4\|IRF9\|JMJD6\|JUND\|KDM1A\|KDM4C\|KDM5A\|KDM5B\|KDM6B\|KLF1\|KLF13\|KLF4\|KLF5\|KLF6\|KLF9\|KMT2A\|LHX2\|LMO2\|LYL1\|MAX\|MAZ\|MBD1\|MBD2\|MBD3\|MED26\|MEF2A\|MEF2C\|MEIS1\|MEIS2\|MIER1\|MITF\|MLLT1\|MNT\|MTA2\|MTA3\|MXI1\|MYB\|MYC\|MYCN\|MYOD1\|NCOA1\|NCOA2\|NCOA3\|NCOR2\|NELFA\|NFATC1\|NFE2\|NFE2L2\|NFIC\|NFKB1\|NFKB2\|NFKBIA\|NKX2-1\|NKX2-2\|NKX3-1\|NME2\|NOTCH1\|NR2C2\|NR2F2\|NR2F6\|NR3C1\|NRIP1\|NSD2\|ONECUT1\|PAX5\|PAX8\|PBX3\|PDX1\|PGR\|PIAS1\|PKNOX1\|POU2F1\|POU2F2\|POU4F2\|POU5F1\|PPARG\|PRDM14\|PROX1\|RAD21\|RARA\|RBPJ\|RCOR1\|RELB\|REST\|RFX1\|RUNX1\|RUNX2\|RUNX3\|RXRA\|SAP30\|SIN3A\|SIN3B\|SIX5\|SMAD2\|SMAD3\|SMAD5\|SMARCA4\|SMARCC1\|SNAI2\|SOX11\|SOX2\|SOX6\|SP1\|SP4\|SPI1\|SPIB\|SREBF1\|SRF\|STAG1\|STAT1\|STAT2\|STAT3\|T\|TAF1\|TAF2\|TAF7\|TAL1\|TBL1X\|TBL1XR1\|TBX21\|TCF12\|TCF3\|TCF7\|TCF7L2\|TEAD1\|TEAD4\|TERF1\|TERF2\|TFAP2A\|TFAP2C\|TFAP4\|TGIF2\|TP53\|TP63\|TP73\|UBTF\|VDR\|YAP1\|YY1\|ZBTB16\|ZBTB7A\|ZEB1\|ZEB2\|ZFP36\|ZFX\|ZHX2\|ZKSCAN1\|ZMIZ1\|ZNF143\|ZNF217\|ZNF24\|ZNF263\|ZNF274\|ZNF384\|ZNF644 |
| GO:0048518 | positive regulation of biological process | 8.55E-05 | 4.88E-02 | 224 | ADNP\|AHR\|AR\|ARID1A\|ARID3A\|ARNT\|ARRB1\|ATF1\|ATF2\|ATF3\|BACH1\|BATF\|BCL11A\|BCL3\|BCL6\|BMI1\|BRCA1\|BRD4\|CBFA2T2\|CBFB\|CDK6\|CDK8\|CDK9\|CDX2\|CEBPB\|CEBPD\|CEBPG\|CHD1\|CHD8\|CIITA\|CREB1\|CREB3L1\|CREBBP\|CREM\|CTBP2\|CTCFL\|CUX1\|DEK\|DPF2\|E2F1\|E2F7\|EBF1\|EED\|EGLN2\|EGR1\|ELF1\|ELF3\|ELK1\|EOMES\|ERG\|ESR1\|ETS1\|ETV1\|ETV4\|ETV6\|EZH1\|EZH2\|FLI1\|FOS\|FOSL1\|FOSL2\|FOXA1\|FOXK1\|FOXK2\|FOXO1\|FOXP1\|FOXP2\|GATA1\|GATA2\|GATA3\|GATA4\|GRHL2\|HCFC1\|HDAC8\|HIF1A\|HMBOX1\|HNF4G\|HSF1\|ID3\|IKZF1\|IRF1\|IRF2\|IRF4\|JMJD6\|JUND\|KDM1A\|KDM4C\|KDM5A\|KDM5B\|KDM6B\|KLF1\|KLF13\|KLF4\|KLF5\|KLF6\|KMT2A\|LHX2\|LMO2\|LYL1\|MAX\|MAZ\|MBD2\|MED26\|MEF2A\|MEF2C\|MEIS1\|MEIS2\|MIER1\|MITF\|MNT\|MTA2\|MTA3\|MYB\|MYC\|MYCN\|MYOD1\|NBN\|NCOA1\|NCOA2\|NCOA3\|NCOR2\|NELFA\|NFATC1\|NFE2\|NFE2L2\|NFIC\|NFKB1\|NFKB2\|NFKBIA\|NKX2-1\|NKX2-2\|NKX3-1\|NME2\|NOTCH1\|NR2C2\|NR2F2\|NR3C1\|NRIP1\|NSD2\|ONECUT1\|PAX5\|PAX8\|PBX3\|PDX1\|PGR\|PHF8\|PIAS1\|PKNOX1\|PML\|POU2F1\|POU2F2\|POU4F2\|POU5F1\|PPARG\|PRDM14\|PROX1\|RAD21\|RARA\|RBPJ\|RELB\|REST\|RUNX1\|RUNX2\|RUNX3\|RXRA\|SIN3A\|SIX5\|SMAD2\|SMAD3\|SMAD5\|SMARCA4\|SMARCC1\|SNAI2\|SOX11\|SOX2\|SOX6\|SP1\|SP4\|SPI1\|SPIB\|SREBF1\|SRF\|STAG1\|STAT1\|STAT2\|STAT3\|SVIL\|T\|TAF1\|TAF2\|TAF7\|TAL1\|TBL1X\|TBL1XR1\|TBX21\|TCF12\|TCF3\|TCF7L2\|TEAD1\|TEAD4\|TERF1\|TERF2\|TFAP2A\|TFAP2C\|TFAP4\|TGIF2\|TP53\|TP63\|TP73\|TRIM22\|TRIM24\|UBTF\|VDR\|YAP1\|YY1\|ZBTB16\|ZBTB7A\|ZEB1\|ZEB2\|ZFP36\|ZFX\|ZMIZ1\|ZNF143\|ZNF24 |

**Supplementary Table 2**

| Tissue | cells |
| --- | --- |
| blood | k562 ocily7 dnd41 mm1s bcell dohh2 sudhl6 karpas422 ocily3 gm12878 gm06990 gm12872 gm19238 gm19239 gm12873 gm12891 gm12870 gm12864 bjab gm20000 gm13977 hbl1 blue1 farage sudhl5 ocily10 ca46 ocily1 namalwa gm15510 gm18951 raji wsudlcl2 pfeiffer gm12801 gm10266 gm10248 granta519 sudhl4 lclgm10861 ramos gm10847 sudhl10 gm18505 gm19193 u2932 bc3 lymphocyte loucy jurkat sudhl2 koptk1 hcc1395 hpball cd8 ccrfcem prima5 rpmi8402 tlymphocyte delta47 fibroblast gm12892 hl60 bcbl1 nb4 gm12869 gm12875 gm19240 gm12866 gm12874 gm19099 lymphoblastoid gm12865 gm12868 gm12867 813 hspc cd4 macrophage mv411 kasumi1 monocyte u937 thp1 nomo1 uae skno1 me1 amlpz12 cal1 cutll1 molt3 prima2 erythroid molm14 mo91 set2 hep ecfc sem gm18526 nalm6 thymocyte dendrite bl41 tsu1621mt amlblast p4936 ncih2171 lcl lncapabl gm13976 cd34 hmsc ncih526 shsy5y shep21 imsc3 ocily19 erythroblast rsa411 ncih929 tf1 kg1 smskcnr smskcn l1236 |
| bone | osteoblast saos2 hfob u2os bone sw1353 |
| breast | t47d breast mda231 sum159 hcc1954 sum149 ab32 whim12 mcf7 mcf10a zr751 mdamb134vi mda157 bt20 mdamb453 mdamb231 bt474 skbr3 sum185 ncih3396 hcc1428 mda436 bt549 hs578t sum1315 hcc2157 imec hmler bpler bpe hme1 |
| cancer cell line | ycc3 |
| endothelial | hmec1 endothelial |
| hESC | esc wibr3 wa09 bgo3 |
| eye | werirb1 retinal |
| fat | asc sgbs |
| female reproductive | ovcar8 ovca429 peo1 ovsaho kuramochi jhos4 ishikawa kb stroma ft246 ft194 ft33 |
| fibroblast | gm00011 gm06170 chrf28811 |
| gut | hct166 caco2 colo741 ht29 gp5d hct116 lovo ls174t hct15 colon ls180 colo320 sw480 rko ovcar3 dld1 colo205 sw620 spleen katoiii ags mkn28 hug1n endoderm |
| heart | ac16 pave |
| hela | hela helab2 |
| huvec | huvec |
| ips | amips6 uteips11 uteips6 uteips7 amips8 uteips4 |
| kidney | 786o cd14 hek293 flp143ha flp76 kidney hek293t hek |
| liver | hepg2 liver huh7 hepatocyte proes lx2 cclp1 hucct1 |
| lung | a549 ncih3122 ncih889 h128 lung ncih1819 ncih524 ncih1299 calu3 wi38 imr90 mrc5 wi38va13 ncih838 ncih2087 hcc95 ncih1703 h1299 ncih2107 ncih441 msto sclc hbe |
| male reproductive | nccit ntera2 nt2d1 |
| mesenchymal | mesenchymal |
| muscle | myotube muscle myoblast rhabdomyosarcoma leiomyoma |
| neuronal | astrocyte sknsh hnsc ipsc neural ngp kelly lan6 pfsk1 gic gbm1a hnpc progenitor glioma npcs renvm daoy ncih295r be2c h54 medulloblastoma sknmc u87mg mpnst |
| oesophagus | kyse150 oe33 kyse70 |
| pancreas | islet pancreas pdac panc1 |
| prostate | lncap du145 vcap patu8988 lhsar prostate c42b pc3 c42 ducap wpmy1 ep156t plhsar 22rv1 rwpe1 |
| skin | keratinocyte hff tb40e epithelial bj nhek hacat a375 ncih82 hmelbraf melanocyte 501mel lp1 mdamb45 hdf nhdf skmel147 dermal wn8532 wm451lu edomips2 |
| stomach | gist snu216 |
| thyroid | tt |
| unknown | mutul |

**Supplementary Table 3**

| tissue | TFs(n) | TFs |
| --- | --- | --- |
| blood | 300 | ADNP AFF4 ARID3A ARNT ATF1 ATF2 ATF3 ATF4 ATF7 ATRX BACH1 BACH2 BATF BCL11A BCL3 BCL6 BCLAF1 BCOR BDP1 BHLHE22 BHLHE40 BMI1 BRCA1 BRD2 BRD3 BRD4 BRF1 CBFB CBX1 CBX2 CBX3 CCNT2 CDK7 CDK8 CDK9 CEBPA CEBPB CEBPD CEBPG CEBPZ CHD1 CHD2 CHD4 CHD7 CIITA CREB1 CREB3 CREB3L1 CREBBP CREM CTCF CTCFL CUX1 DDX20 DEAF1 DIDO1 DPF2 E2F4 E2F5 E2F6 EBF1 EED EGR1 ELF1 ELK1 EP300 EPAS1 ERG ESRRA ETS1 ETV1 ETV6 EZH2 FANCL FLI1 FOS FOSL1 FOXJ2 FOXK2 FOXM1 FOXO1 FOXP1 GABPA GATA1 GATA2 GATA3 GFI1B GTF2B GTF2F1 GTF3C2 HCFC1 HDAC1 HDAC2 HDAC6 HDAC8 HDGF HIF1A HINFP HMBOX1 HMGN3 HOXA9 HSF1 ID3 IKZF1 ILK IRF1 IRF2 IRF3 IRF4 IRF9 JMJD1C JUN JUNB JUND KAT2B KDM1A KDM4B KDM5B KDM6B KLF1 KLF13 KLF5 KMT2A KMT2B L3MBTL2 LDB1 LMO2 LYL1 MAF MAFF MAFG MAFK MAX MAZ MED1 MEF2A MEF2B MEF2C MEIS1 MEIS2 MIER1 MITF MLLT1 MLLT3 MTA2 MTA3 MXI1 MYB MYC MYCN MYNN NBN NCOA1 NCOR1 NCOR2 NEUROD1 NFATC1 NFE2 NFE2L1 NFE2L2 NFIC NFKB1 NFKB2 NFRKB NFYA NFYB NIPBL NONO NOTCH1 NR1H3 NR2C2 NR2F1 NR2F2 NR3C1 NR4A1 NRF1 NSD2 OCA2 PAX5 PAX8 PBX2 PBX3 PCGF2 PHF8 PKNOX1 PML POU2F2 POU5F1 PPARG PRKDC PTTG1 PYGO2 RAD21 RAD51 RARA RB1 RBBP5 RBP2 RBPJ RCOR1 RELA RELB REST RFX5 RNF2 RUNX1 RUNX1T1 RUNX2 RUNX3 RXRA SAP30 SATB1 SETDB1 SIN3A SIN3B SIRT6 SIX5 SMAD1 SMAD2 SMAD5 SMARCA4 SMARCB1 SMC1A SMC3 SOX11 SOX6 SP1 SP2 SPI1 SPIB SREBF1 SREBF2 SRF STAG1 STAT1 STAT2 STAT3 STAT5A STAT5B SUZ12 SVIL TAF1 TAF7 TAL1 TARDBP TBL1XR1 TBP TBX21 TCF12 TCF3 TCF7 TCF7L2 TEAD2 TEAD4 TERF1 TERF2 TFAP4 TFDP1 THAP1 TP53 TRIM22 TRIM24 TRIM28 TSC22D4 TWIST1 UBTF USF1 USF2 VDR WRNIP1 YY1 ZBED1 ZBTB11 ZBTB33 ZBTB7A ZC3H11A ZEB1 ZEB2 ZFP36 ZFX ZHX1 ZKSCAN1 ZMIZ1 ZNF143 ZNF175 ZNF197 ZNF207 ZNF24 ZNF263 ZNF274 ZNF318 ZNF384 ZNF584 ZNF589 ZNF639 ZNF644 ZNF740 ZNF766 ZNF83 ZZZ3 |
| bone | 24 | ARNTL BRD4 CEBPB CLOCK CRY1 CTCF EP300 FOSL2 FOXM1 HES1 HSF1 JUND MED26 MYC NR3C1 RAD51 RUNX1 SETDB1 SIRT3 TEAD1 TP53 TP73 TRIM28 ZMYND11 |
| breast | 108 | AHR AR ARNT BCL11A BRCA1 BRD4 CARM1 CDK2 CDK9 CEBPB CEBPG CHD8 CREBBP CTBP1 CTCF CUX1 DPF2 E2F1 E2F4 EGLN2 EGR1 ELF1 ELF5 ELK1 EP300 ERG ESR1 ESR2 ESRRA FOS FOSL1 FOSL2 FOXA1 FOXK2 FOXM1 GABPA GATA3 GTF2F1 HCFC1 HDAC2 HIF1A HOXB7 HSF1 JUN JUND KDM5A KDM5B KLF4 KLF9 MAFK MAX MAZ MBD2 MBD3 MED1 MLLT1 MNT MTA1 MTA2 MYC NCOA1 NCOA2 NCOA3 NELFA NONO NR2F1 NR2F2 NR3C1 NR5A2 NRF1 NRIP1 PALB2 PGR PML PRKDC RAC3 RAD21 RCOR1 RELA REST RFX5 RUNX1 SIN3A SMARCA4 SNAPC1 SNAPC4 SREBF1 SRF STAG1 STAT3 TAF1 TCF12 TCF7L2 TEAD4 TFAP2A TFAP2C TLE3 TP53 TP63 UCREBBP XBP1 YAP1 ZBTB11 ZKSCAN1 ZNF143 ZNF165 ZNF217 ZNF592 |
| cancer cell line | 3 | GATA4 GATA6 KLF5 |
| endothelial | 12 | CTCF E2F1 EZH2 FOS FOXM1 GATA2 JUN MAX MBD2 MYBL2 MYC NRF1 |
| hESC | 89 | ATF2 ATF3 BACH1 BCL11A BRCA1 BRD4 CEBPB CHD1 CHD2 CHD7 CREB1 CTBP2 CTCF E2F6 EGR1 ELK1 EOMES EP300 ETS1 EZH2 FOSL1 FOSL2 FOXA2 FOXO1 FOXP1 GABPA GATA6 GTF2F1 HDAC2 HDAC6 HNF1B JUN JUNB JUND KDM4A KDM5A KDM5B LEF1 MAFK MAX MED1 MXI1 MYC NANOG NIPBL NRF1 ONECUT1 PDX1 PGR PHF8 POU5F1 PRDM14 RAD21 RBBP5 REST RFX5 RNF2 RXRA SAP30 SIN3A SIRT6 SIX5 SMAD1 SMAD2 SMAD3 SMAD4 SOX2 SP1 SP2 SP4 SRF STAT3 SUZ12 T TAF1 TAF2 TAF7 TBP TCF12 TEAD4 TP53 TRIM28 UBTF USF1 USF2 YY1 ZBTB16 ZNF143 ZNF274 |
| eye | 7 | CTCF E2F4 LHX2 MITF OTX2 PAX6 ZNF92 |
| fat | 8 | BRD4 CEBPA CTCF EBF1 MED1 NR1H3 PPARG RELA |
| female reproductive | 26 | CDK6 CEBPB CREB1 CTCF CTCFL EGR1 EP300 ESR1 FOXA1 FOXM1 GRHL2 MAX NFIC NR2F2 NR3C1 PAX8 RAD21 RELA REST SRF TAF1 TCF12 TEAD4 USF1 YY1 ZBTB7A |
| fibroblast | 7 | CTCF GATA1 MEIS1 RAD21 STAG1 TAL1 TP53 |
| gut | 61 | AFF4 ATF3 BRD1 BRD4 CBX3 CDK8 CDK9 CDX2 CEBPB CREBBP CTCF CTNNB1 EGR1 ELF1 ELL ELL2 FOSL1 FOXA2 GATA4 GATA6 GRHL2 HEXIM1 HNF4A HSF1 ICE1 ICE2 JUND KLF5 MAX MED1 MYC NCOA1 NCOR2 NIPBL NR1H2 PPARG PROX1 RAD21 REST RFX2 SIN3A SMAD2 SMAD3 SMAD4 SMC3 SOX9 SP1 SRF TAF3 TCF4 TCF7L2 TEAD4 TFAP4 TOP1 TP53 TRIM28 USF1 VDR YY1 ZBTB33 ZC3H8 |
| heart | 2 | MYC RELA |
| hela | 80 | AFF4 BDP1 BRCA1 BRD4 BRF1 CEBPB CHD1 CHD2 CTCF DDX5 DEK E2F1 E2F4 E2F6 ELK1 ELK4 ELL2 EP300 EZH2 FOS FOXM1 GABPA GATAD1 GLYR1 GTF2B GTF2F1 GTF3C2 HCFC1 HMBOX1 IRF3 JMJD6 JUN JUND KAT2A KDM1A MAFF MAFK MAX MAZ MBD2 MXI1 MYC NFYA NFYB NR2C2 NR2F2 NR3C1 NRF1 PHF8 PRDM1 RAD21 RCOR1 RELA REST RFX5 SFMBT1 SMARCA4 SMARCB1 SMARCC1 SMARCC2 SMC3 SREBF2 STAT1 STAT3 SUPT20H TAF1 TBP TCF7L2 TERF2 TRRAP UBN1 USF2 YY1 ZFP36 ZHX1 ZKSCAN1 ZMYND11 ZNF143 ZNF274 ZZZ3 |
| huvec | 11 | BMPR1A BRD2 BRD3 BRD4 ETS1 HIF1A MEF2C NFATC1 PGR PPARG RELA |
| ips | 1 | JARID2 |
| kidney | 35 | AFF4 ASXL1 BAHD1 BRD2 BRD3 BRD4 CBX2 CBX4 CDK9 CTCF CXXC4 DUX4 ELK4 EPAS1 EWSR1 FOXK1 FOXM1 GSPT2 HIF1A IRF1 JMJD6 KLF3 RNF2 RYBP SIX2 SP1 SP2 STAT1 TBL1X TCF7L2 TRIM28 USP7 ZNF143 ZNF263 ZNF644 |
| liver | 111 | ARID1A ARID1B ARID2 ARID3A ATF1 ATF3 ATF4 BACH1 BHLHE40 BRCA1 CBX1 CEBPA CEBPB CEBPD CEBPZ CHD2 CREB1 CTCF CUX1 DNMT3B EED ELF1 EP300 ESRRA ETV4 EZH1 EZH2 FOS FOSL2 FOXA1 FOXA2 FOXK2 GABPA GATA4 HBP1 HCFC1 HDAC2 HHEX HNF4A HNF4G HSF1 IKZF1 IRF3 JUN JUND KAT2B MAFF MAFK MAX MAZ MBD1 MBD4 MXI1 MYBL2 MYC NFE2 NFE2L2 NFIC NR2C2 NR2F2 NR2F6 NR3C1 NRF1 ONECUT1 PBX2 PPARGC1A RAD21 RCOR1 REST RFX1 RFX5 RXRA SIN3A SIN3B SMAD3 SMARCB1 SMC3 SOX13 SP1 SP2 SREBF1 SREBF2 SRF SSRP1 STAG1 SUZ12 TAF1 TAL1 TBL1XR1 TBP TCF12 TCF25 TCF7L2 TEAD1 TEAD4 TFAP4 TGIF2 TP53 USF1 USF2 VDR YAP1 YY1 ZBTB33 ZBTB7A ZEB1 ZHX2 ZKSCAN1 ZNF143 ZNF175 ZNF384 |
| lung | 75 | ASCL1 ATF3 BACH1 BCL3 BDP1 BHLHE40 BRCA1 BRD4 BRF2 CEBPB CHD1 CHD2 CREB1 CTCF E2F6 E2F7 EHF ELF1 ELK1 EP300 ESRRA ETS1 FOSL2 FOXA1 FOXA2 GABPA GATA3 GRHL2 GTF2B HIF1A HMBOX1 HSF1 JARID2 JUN JUND KAT2B MAFK MAX MAZ MXI1 MYC NEUROD1 NFE2L2 NKX2-1 NME2 NR2C2 NR2F2 NR3C1 PBX3 POU2F1 RAD21 RCOR1 RELA REST RFX5 SIN3A SIX5 SMAD3 SP1 SREBF1 SREBF2 TAF1 TCF12 TEAD1 TEAD4 TERF2 TP53 TP63 USF1 USF2 YAP1 YY1 ZBTB33 ZC3H11A ZFP36 |
| male reproductive | 8 | CBFA2T2 DDX5 POU5F1 PRDM14 RNF2 SUZ12 YY1 ZNF274 |
| mesenchymal | 1 | E2F1 |
| muscle | 5 | CTCF EZH2 MED1 MYOD1 PGR |
| neuronal | 47 | ATRX BRD4 CHD8 CTCF ELF1 EP300 EZH2 FLI1 FOSL2 FOXM1 FOXP1 FOXP2 GABPA GATA3 JUND KLF4 KLF9 MAML3 MAX MED1 MEF2A MXI1 MYC MYCN NFIC NRF1 PBX3 POU2F2 POU4F2 POU5F1 RAD21 RARA RBPJ REST RFX5 RXRA SIN3A SMC3 SOX2 TAF1 TCF12 TEAD4 TWIST1 USF1 YY1 ZBTB33 ZFX |
| oesophagus | 4 | FOXM1 KDM4C SOX2 TP63 |
| pancreas | 18 | CTCF ELF3 ETS1 FOXA1 FOXA2 HNF1B IRF1 KLF4 KLF6 MAFB NKX2-2 NKX3-1 PDX1 REST SIN3A TCF7L2 TEAD1 ZEB1 |
| prostate | 40 | AR ARRB1 ASH2L CHD1 CREB1 CSNK2A1 CTBP1 CTBP2 CTCF DAXX E2F1 ERG ESR1 ETS1 ETV1 EZH2 FOXA1 FOXP1 GABPA GATA2 GATA6 HDAC1 HDAC2 HDAC3 HOXB13 JUND NKX3-1 NR3C1 PIAS1 PPARG RELA RUNX1 RUNX2 SUZ12 TCF7L2 TFAP4 TOP1 TP63 TRIM24 VDR |
| skin | 34 | AR ASCL1 BRD4 CDK7 CDK9 CTCF EZH2 HSF1 JARID2 JUNB KDM1A KLF4 MAF MAFB MITF MYC NFE2L2 NFKBIA POU5F1 RBPJ RCOR1 RELA RNF2 SETDB1 SMARCA4 SNAI2 SND1 SOX10 SOX2 TFAP2C TP53 TP63 TRIM28 ZNF750 |
| stomach | 2 | ETV1 TEAD4 |
| thyroid | 2 | SOX2 TP63 |
